# Aryl hydrocarbon receptor nuclear translocator (ARNT) isoforms control lymphoid cancer cell proliferation through differentially regulating tumor suppressor p53 activity

**DOI:** 10.18632/oncotarget.7539

**Published:** 2016-02-20

**Authors:** Kacie A. Gardella, Israel Muro, Gloria Fang, Krishnakali Sarkar, Omayra Mendez, Casey W. Wright

**Affiliations:** ^1^ Division of Pharmacology and Toxicology, and the Center for Molecular and Cellular Toxicology, College of Pharmacy, The University of Texas at Austin, Austin, TX, USA; ^2^ Institute for Cellular and Molecular Biology, The University of Texas at Austin, Austin, TX, USA

**Keywords:** alternative splicing, ARNT, RelB, p53, lymphoid malignancies

## Abstract

The aryl hydrocarbon receptor nuclear translocator (ARNT) is involved in xenobiotic and hypoxic responses, and we previously showed that ARNT also regulates nuclear factor-κB (NF-κB) signaling by altering the DNA binding activity of the RelB subunit. However, our initial study of ARNT-mediated RelB modulation was based on simultaneous suppression of the two ARNT isoforms, isoform 1 and 3, and precluded the examination of their individual functions. We find here that while normal lymphocytes harbor equal levels of isoform 1 and 3, lymphoid malignancies exhibit a shift to higher levels of ARNT isoform 1. These elevated levels of ARNT isoform 1 are critical to the proliferation of these cancerous cells, as suppression of isoform 1 in a human multiple myeloma (MM) cell line, and an anaplastic large cell lymphoma (ALCL) cell line, triggered S-phase cell cycle arrest, spontaneous apoptosis, and sensitized cells to doxorubicin treatment. Furthermore, co-suppression of RelB or p53 with ARNT isoform 1 prevented cell cycle arrest and blocked doxorubicin induced apoptosis. Together our findings reveal that certain blood cancers rely on ARNT isoform 1 to potentiate proliferation by antagonizing RelB and p53-dependent cell cycle arrest and apoptosis. Significantly, our results identify ARNT isoform 1 as a potential target for anticancer therapies.

## INTRODUCTION

Hematological malignancies, like many other cancers, often manifest genetic amplification events that alter gene expression and allow unrestrained survival and proliferation [1]. For example, MM tumors, an age-dependent tumor of bone marrow plasma cells, often exhibit a gain of chromosome 1q21, which is a prognostic indicator of poor overall survival [2, 3]. An analogous illustration is seen in the childhood-prevalent T cell cancer, ALCL, where cytogenetic analysis of ALCL tissues and ALCL-derived cell lines revealed that a common DNA copy number gain was observed in the chromosome 1q21 region [4, 5]. While amplification of *CKS1B*, a gene within the 1q21 region, has been shown to be important in disease progression, the contribution of other genes within this amplified region is not clear [6].

*ARNT* is a gene contained within the amplified region of chromosome 1q (1q21.3) and, in MM patients, high levels of *ARNT* expression are associated with an unfavorable outcome [7]. Based on these observations, in this report we examined whether ARNT regulated the proliferation and survival of malignant blood cells. Also known as hypoxia inducible factor-1β (HIF-1β), ARNT is a member of the basic helix-loop-helix/Per-ARNT-Sim family of transcription factors and predominantly heterodimerizes with the aryl hydrocarbon receptor (AHR) or HIF-1α [8-10]. Deregulation of AHR and HIF-1α activity can promote various disease states including cancer proliferation, and ARNT has been shown to be essential in supporting these pathophysiological characteristics [11-15].

Independent of its role in AHR and HIF signaling, ARNT has also been reported to support the proliferation and survival of a number of tumor cell lines by regulating various cellular processes [16-19]. In fact, we have found that ARNT inhibits NF-κB, which is a transcription factor that drives the expression of pro-survival and mitogenic factors [20]. Not surprisingly, 17% of MM tumors and 40% of human MM cell lines (HMCL) exhibit constitutive NF-κB signaling [21-24]. Moreover, multiple studies have demonstrated that aberrant NF-κB activity is vital to the proliferation and survival of these cancerous cells [21-24]. NF-κB signaling is accomplished through differential dimerization of five subunits known as RelA, RelB, c-Rel, p50/p105 and p52/p100 [25]. In a previous report, we found that ARNT promoted RelB DNA binding to block the activity of RelA-p50 dimers, i.e. in the absence of ARNT protein, RelB DNA binding was decreased, RelA DNA binding was increased, and NF-κB activity was augmented [20]. Though these results predict that an amplification of ARNT protein would inhibit NF-κB signaling, ARNT is expressed as two alternatively spliced isoforms and our previous study did not explore isoform specific functions [26].

The ARNT isoforms differ by the exclusion or inclusion of a short N-terminal exon that provides isoform 1 with an extra 15 amino acids as compared to isoform 3. ARNT isoform 1 and 3 are highly conserved. For example, ARNT isoform 1 and 3 both share ∼92% homology, at the amino acid level, with murine Arnt-a and b, respectively, including 100% homology between the 15 amino acid stretch that defines ARNT isoform 1. However, specific ARNT isoform 1 and 3 function has only been evaluated by a single study that identified a casein kinase 2 phosphorylation site within the extra 15 amino acids of isoform 1, imparting regulation of DNA binding [27]. Since many of the experiments in this previous study utilized *in vitro* and recombinant protein assays, we sought to explore ARNT isoform activities in intact cells. Here we find that while normal lymphocytes exhibit equal levels of isoform 1 and 3, lymphoid malignancies express ARNT isoform 1 almost exclusively. This observation led us to hypothesize that ARNT isoform 1 provides a proliferation advantage to cancer cells. Through targeted suppression, we uncover a requirement for ARNT isoform 1 in sustaining proliferation and supporting cell survival. We observe that in the absence of ARNT isoform 1, malignant blood cells exhibit slowed proliferation and increased levels of cell death. Unexpectedly, the manifestations of these phenotypes require RelB and p53 activity but appear to be independent of NF-κB signaling. Importantly, there are examples of deregulated alternative splicing events that aid the proliferation of cancers, and our findings suggest that a shift to ARNT isoform 1 production may be critical to the oncogenesis of blood cell derived malignancies [28, 29].

## RESULTS

### *ARNT* amplification is prevalent in HMCLs and ALCL cell lines

**Figure 1:**
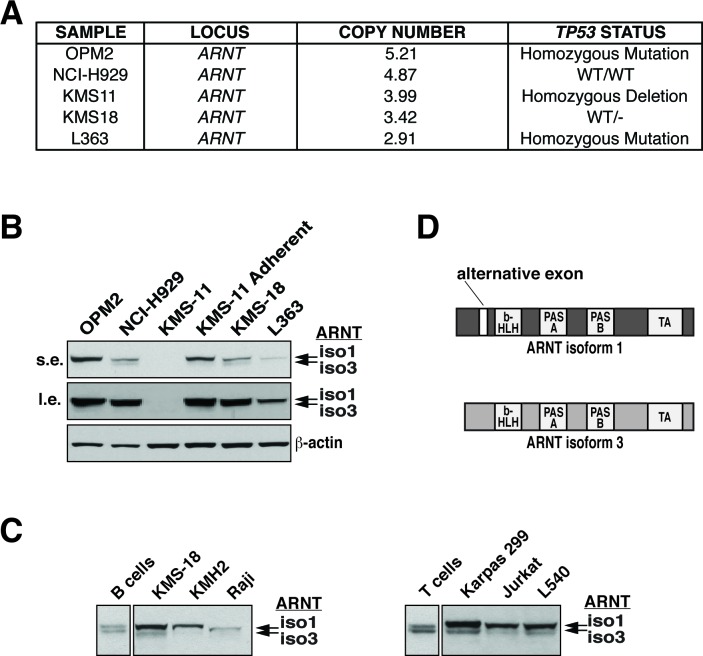
ARNT amplification in HMCLs and expression in lymphoid malignancies **A.** *ARNT* copy number, along with *TP53* status, is shown for select HMCLs. **B.** ARNT protein levels in the indicated HMCLs were analyzed by immunoblotting (s.e., short exposure; l.e., long exposure). β-actin serves as a loading control. **C.** Normal human B and T cells were isolated from whole blood by magnetic selection (Miltenyi Biotec), and ARNT protein levels were analyzed by immunoblotting. These were compared to ARNT immunoblots of whole cell lysates obtained from the indicated B and T cell cancer cell lines. **D.** Schematic representation of ARNT isoforms 1 and 3; b-HLH, basic helix-loop-helix; PAS, Per-ARNT-Sim; TA, transactivation.

**Figure 2:**
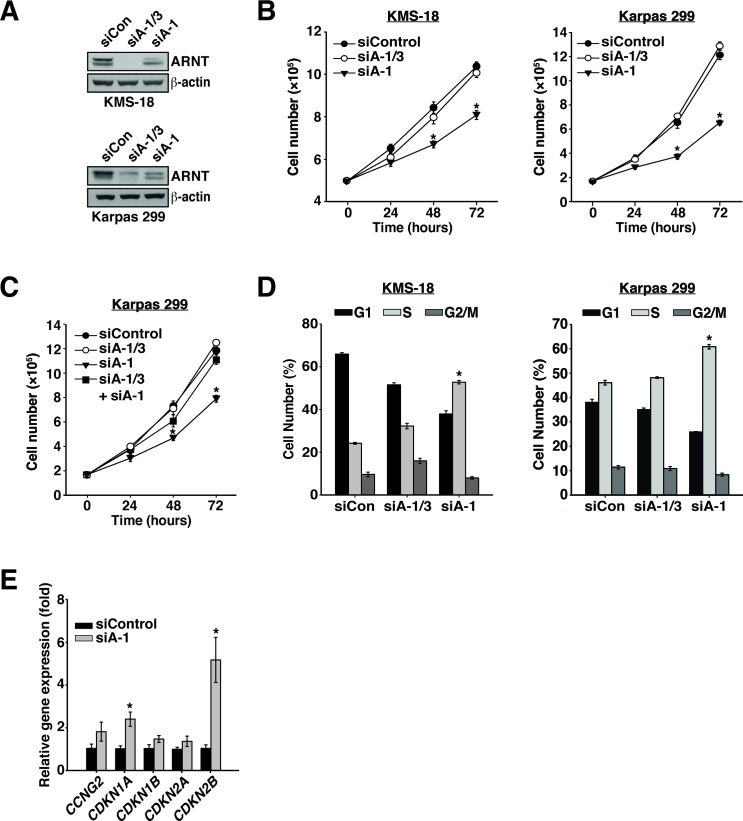
ARNT isoform 1 promotes cancer cell proliferation through cell cycle regulation **A.** KMS-18 and Karpas 299 cells were electroporated with a control siRNA (siControl), an siRNA targeting both isoforms (siA-1/3), or an siRNA targeting isoform 1 specifically (siA-1). Protein levels were assessed by immunoblotting 48 hours post-transfection. β-actin serves as a loading control. **B.** Twenty-four hours after electroporation with siControl, siA-1/3, or siA-1, KMS-18 and Karpas 299 cells were normalized and counted every 24 hours for 72 hours. **C.** Twenty-four hours after electroporation with siControl, siA-1/3, siA-1, or siA-1/3 + siA-1, Karpas 299 cells were normalized and counted every 24 hours for 72 hours. **D.** KMS-18 and Karpas 299 cells, transfected as in **A.**, were stained 48 hours post-transfection with PI to evaluate cell cycle profiles by flow cytometry. **E.** Total RNA was collected from Karpas 299 cells treated with siControl or siA-1 for 48 h and subjected to reverse transcription followed by qPCR analysis of cell cycle inhibitor gene expression. *p < 0.001 compared to siControl.

Examination of various HMCLs by array comparative genomic hybridization (aCGH) displayed frequent focal amplifications of chromosome 1q21 leading to multiple copies of the *ARNT* locus (Figures 1A and S1A). ARNT protein levels mostly correlated with copy number except for KMS-11 cells, which exhibit unstable ARNT protein over time in culture (Figures 1B and S1B). Interestingly, analysis of the Oncomine database revealed that ARNT is amplified in a number of diverse cancers (Figure S1C). To further ascertain the effects of *ARNT* amplification on ARNT protein levels, we compared human primary B and T cells with human B and T cell cancers by immunoblot analysis. Two protein entities were detected with the ARNT antibody, which appeared at equal levels in naïve B and T cells, whereas in lymphoid cancers increased levels of the higher molecular weight form were observed (Figure 1B and 1C). ARNT undergoes a variety of modifications, and it was unclear whether the two products resulted from protein processing, post-translational alterations, or differential splicing. Since the ARNT isoforms differ by only 15 amino acids, we speculated that the observed proteins were ARNT isoform 1 and 3 (Figure 1D). Our supposition was confirmed by introducing an isoform 1-specific siRNA duplex (siA-1) into KMS-18 (a HMCL) or Karpas 299 (an ALCL cell line with reported amplification of the 1q21 region), which only suppressed the higher molecular weight product (Figure 2A) [5]. In contrast, targeting of a common sequence in isoform 1 and 3 (siA-1/3) resulted in suppression of both products (Figure 2A). These results confirm the expression of ARNT isoform 1 and 3 and demonstrate that B and T cell cancers have increased isoform 1 levels.

To gain insight into the elevated levels of ARNT isoform 1, we analyzed the epigenomic profile of the *ARNT* locus using data sets derived from reference genomes by the NIH Roadmap Epigenomics Consortium [30]. Interestingly, the resulting profile revealed a conspicuous pattern of acetylated and methylated histones centered on the alternative exon 5 in primary lymphoid cells, suggesting highly relaxed and accessible DNA in this region (Figure S2). Conversely, cancer cell epigenomes showed loss of these exon 5-associated histone marks, suggesting a lymphoid specific means of epigenetically regulating the exclusion of exon 5 (Figure S2) [31].

### ARNT isoform 1 confers a proliferation advantage to HMCL and ALCL cells

Given the increased presence of ARNT isoform 1 we hypothesized that isoform 1 imparts an advantage to cancer cell proliferation. This notion was confirmed by suppression of ARNT isoform 1 in KMS-18 cells or Karpas 299 cells, which resulted in significant proliferation reduction (Figure 2B). Notably, simultaneous suppression of both isoforms had no effect on proliferation, suggesting that the decrease in proliferation observed when ARNT isoform 1 was suppressed results from isoform 3 activity. Moreover, the observed defect in proliferation was specific to suppression of ARNT isoform 1, given that combining siA-1/3 with siA-1 did not affect cell proliferation (Figure 2C).

ARNT has been reported to drive the expression of the cell cycle inhibitor *CDKN2B* [32]. Thus, we investigated whether the proliferation deficit associated with the suppression of ARNT isoform 1 was a result of changes in the cell cycle. Remarkably, reduction of ARNT isoform 1 in KMS-18 or Karpas 299 cells triggered S phase cell cycle arrest (Figure 2D). In addition, analysis of a panel of cell cycle inhibitors showed significant upregulation of *CDKN1A* and *CDKN2B* expression, of which the product of *CDKN1A*, p21^WAF1^, has been shown to block S phase cell cycle progression (Figure 2E) [33, 34]. Together these data suggest that in the absence of ARNT isoform 1, ARNT isoform 3 induces the expression of cell cycle inhibitors to impede cancer proliferation.

### RelB participates in ARNT isoform 1 and 3-mediated proliferation regulation

**Figure 3:**
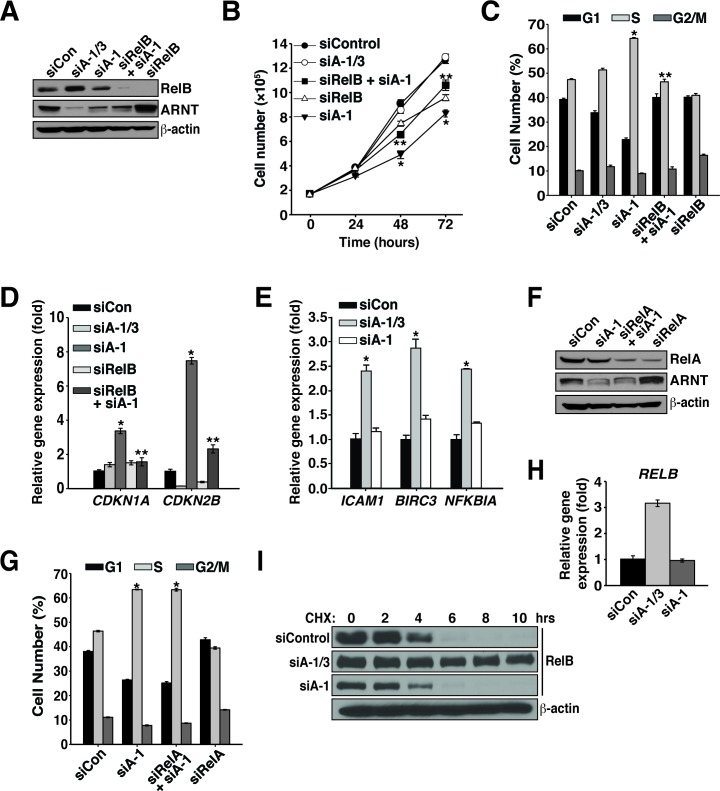
Proliferation regulation by ARNT isoform 1 and 3 involves RelB **A.** Karpas 299 cells were electroporated with siControl, siA-1/3, siA-1, siRelB and siA-1, or siRelB, and 48 hours later, protein levels were analyzed by immunoblotting with the antibodies shown. **B.** Cell proliferation was measured in Karpas 299 cells, after introduction of siControl, siA-1/3, siA-1, siRelB and siA-1, or siRelB, by counting them every 24 hours for 72 hours. **C.** Cell cycle analysis using propidium iodide was performed 48 hours after transfection of Karpas 299 cells with siControl, siA-1/3, siA-1, siRelB and siA-1, or siRelB. **D.** qPCR analysis of *CDKN1A* and *CDKN2B* gene expression in Karpas 299 cells 48 hours after electroporation with the indicated siRNAs. **E.** qPCR analysis of noncanonical NF-κB target genes using total RNA isolated from Karpas 299 cells electroporated as in **A. F.** Karpas 299 cells were electroporated with siControl, siA-1, siRelA and siA-1, or siRelA and protein levels were analyzed 48 hours later by immunoblotting. β-actin serves as a loading control. **G.** Cell cycle analysis of Karpas 299 cells treated as in **F. H.** Forty-eight hours post-transfection, qPCR analysis of *RELB* expression was performed using total RNA isolated from Karpas 299 cells that had been electroporated with siControl, siA-1/3, or siA-1. **I**. Karpas 299 cells electroporated with siControl, siA-1/3, or siA-1 were treated with 100 μg/mL cycloheximide at 60 hours post-transfection and lysed at the indicated time points. Cell lysates were analyzed by immunoblot for changes in RelB half-life. The β-actin immunoblot corresponds to the siControl sample and is similar to β-actin levels from the siA-1/3 and siA-1. *p < 0.001 compared to siControl. **p < 0.001 compared to siA-1.

Since we previously found that loss of the ARNT isoforms leads to modification of RelB DNA binding, we sought to investigate whether the ARNT isoforms depend on RelB for regulating cancer cell proliferation. To test this hypothesis, RelB protein was suppressed alone (siRelB) or in combination with ARNT isoform 1 (Figure 3A). Because KMS-18 cells are dependent on RelB for survival and proliferation, we were unable to assess RelB in KMS-18 cells and instead focused on Karpas 299 cells where suppression of RelB only partially affected cell proliferation (Figure 3B) [23]. Interestingly, co-suppression of RelB and ARNT isoform 1 significantly rescued the defects in proliferation, cell cycle progression, and expression of cell cycle inhibitors (Figure 3B-3D).

Given the requirement for RelB in preventing proliferation after depletion of ARNT isoform 1, we investigated whether NF-κB activity was affected after suppression of ARNT isoform 1. In agreement with our previous study, suppression of both ARNT isoforms augmented the expression of several NF-κB target genes (Figure 3E) [20]. However, to our surprise, reducing ARNT isoform 1 had no effect on NF-κB activity toward the target genes tested, indicating that while RelB is essential for triggering cell cycle arrest, NF-κB signaling, *per se*, has no role (Figure 3E). To further confirm the lack of involvement of NF-κB activity, RelA was suppressed (siRelA) with ARNT isoform 1. As expected, cell cycle analysis showed that RelA was dispensable for inducing S phase cell cycle arrest (Figure 3F and 3G). Thus, the cell cycle arrest signal that is induced after depletion of ARNT isoform 1 requires RelB but does not depend on NF-κB signaling.

### ARNT isoforms 1 and 3 regulate RelB expression and protein stability

Analysis of *RELB* transcription in cells devoid of both ARNT isoforms resulted in elevated levels of transcription (Figure 3H) [20]. However, reduction of ARNT isoform 1 fails to alter *RELB* gene expression, which instead suggests a change in protein stability (Figure 3H). To explore if the ARNT isoforms regulate the stability of RelB protein, Karpas 299 cells were treated with cycloheximide after transfecting with siControl, siA-1/3, or siA-1. Immunoblot analysis of control treated cells demonstrated that under normal conditions RelB has a half-life of ∼5 hours (Figure 3I). Interestingly, suppression of both ARNT isoforms stabilized RelB by increasing the protein half-life to over 10 hours. However, suppression of ARNT isoform 1 modestly diminished RelB stability (Figure 3I). These data indicate that ARNT isoforms contribute to RelB expression and turnover and also demonstrate that different ARNT isoform ratios produce opposing effects on the regulation of RelB protein.

### ARNT isoforms 1 and 3 control p53 protein stability and activity

**Figure 4:**
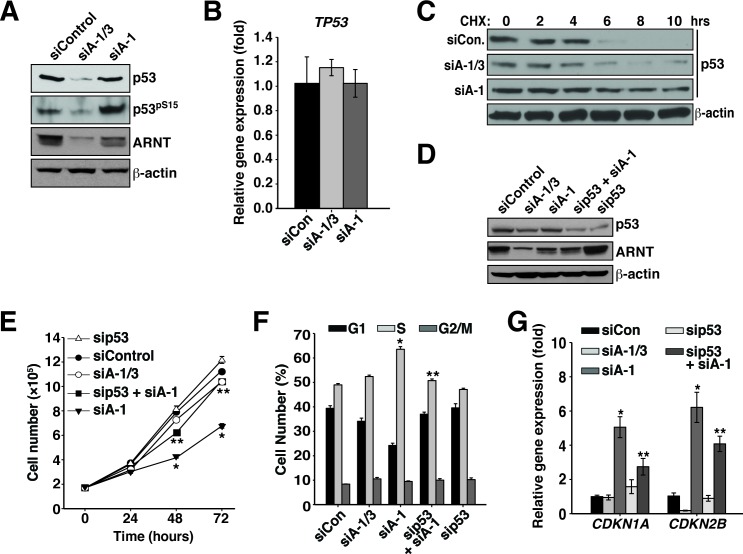
ARNT isoforms 1 and 3 control p53 protein stability and activity in Karpas 299 cells **A.** Immunoblot analysis of p53 protein levels and phosphorylation state in Karpas 299 cells that were electroporated with siControl, siA-1/3, or siA-1. β-actin serves as a loading control. **B.** qPCR analysis of *TP53* expression using total RNA isolated from Karpas 299 cells that had been electroporated with siControl, siA-1/3, or siA-1. **C.** Karpas 299 cells electroporated with siControl, siA-1/3, or siA-1 were treated with 100 μg/mL cycloheximide at 60 hours post-transfection and lysed at the indicated time points. Cell lysates were analyzed by immunoblot for changes in p53 half-life. The β-actin immunoblot corresponds to the siControl sample and is similar to β-actin levels from the siA-1/3 and siA-1. **D.** Karpas 299 cells were electroporated with siControl, siA-1/3, siA-1, sip53 and siA-1, or sip53 and lysed 48 hours later. Protein levels were analyzed by immunoblotting for p53 and ARNT. β-actin serves as a loading control. **E.** Cell proliferation was measured in Karpas 299 cells after introduction of siControl, siA-1/3, siA-1, sip53 and siA-1, or sip53, by counting them every 24 hours for 72 hours. **F.** Karpas 299 cells were electroporated with siControl, siA-1/3, siA-1, sip53 and siA-1, or sip53. Forty-eight hours post-transfection the cell cycle was analyzed by PI staining and flow cytometry. **G.** qPCR analysis of *CDKN1A* and *CDKN2B* gene expression in Karpas 299 cells electroporated with siControl, siA-1/3, siA-1, sip53 and siA-1, or sip53.

RelB has been shown to suppress cell senescence and promote tumorigenesis by disrupting the stability of the tumor suppressor p53 [35]. These findings, coupled with the fact that the ARNT isoforms modulate RelB protein stability and the expression of cell cycle inhibitors, led us to postulate that the ARNT isoforms influence p53 activity. While Karpas 299 cells have been reported to have a point mutation in p53 that changes arginine 273 to cysteine, it has been shown that the R273C p53 mutant is stable and retains the ability to bind DNA [36, 37]. Furthermore, data presented here, in combination with our previous study, support a wildtype function for p53 in Karpas 299 cells [38]. To investigate our hypothesis we assessed p53 activity in Karpas 299 cells after introduction of siControl, siA-1/3, and siA-1. Strikingly, we observed that in the absence of both ARNT isoforms, p53 protein levels are significantly reduced (Figure 4A). Additionally, suppression of ARNT isoform 1 resulted in increased levels of serine 15 phosphorylated p53, which is an activation marker (Figure 4A). Quantitative PCR revealed that the reduction in p53 protein was not a consequence of transcriptional control since p53 expression was similar between siControl, siA-1/3, and siA-1 (Figure 4B). Interestingly, despite no changes in *TP53* gene expression, suppression of ARNT isoform 1 resulted in dramatic stabilization of p53 protein (Figure 4C). These findings suggest that the S phase arrest associated with lowering ARNT isoform 1 occurs through activation of p53. To examine this notion, cell cycle analysis was performed on cells with reduced levels of ARNT isoform 1 and p53. As predicted, decreasing p53 protein levels together with ARNT isoform 1 reversed the cell cycle arrest phenotype and significantly lowered *CDKN1A* and *CDKN2B* expression (Figure 4D-4G).

**Figure 5:**
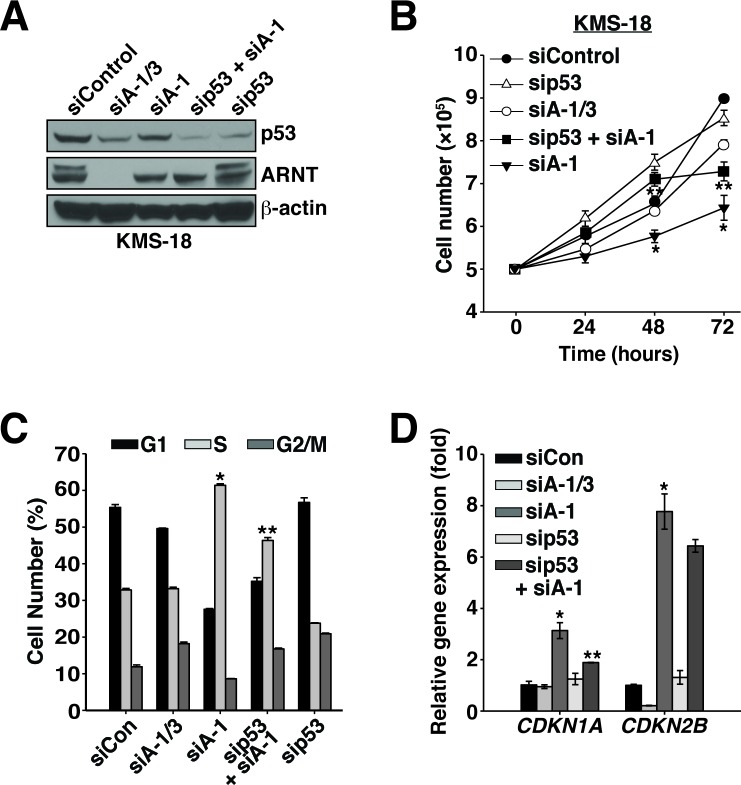
ARNT isoforms 1 and 3 control p53 protein stability and activity in KMS-18 cells **A.** KMS-18 cells were electroporated with siControl, siA-1/3, siA-1, sip53 and siA-1, or sip53, and protein levels were analyzed 64 hours post-transfection by immunoblotting. β-actin serves as a loading control. **B.** Cell proliferation was measured in KMS-18 cells after introduction of siControl, siA-1/3, siA-1, sip53 and siA-1, or sip53, by counting them every 24 hours for 72 hours. **C.** KMS-18 cells were electroporated with siControl, siA-1/3, siA-1, sip53 and siA-1, or sip53. Sixty-four hours post-transfection the cell cycle was analyzed by PI staining and flow cytometry. **D.** qPCR analysis of *CDKN1A* and *CDKN2B* gene expression in Karpas 299 cells 48 hours post-transfection with siControl, siA-1/3, siA-1, sip53 and siA-1, or sip53. *p < 0.001 compared to siControl. **p < 0.001 compared to siA-1.

Cooperation between p53 and ARNT was also examined in KMS-18 cells, which encode wildtype p53 (Figure 1A) [39]. Remarkably, ARNT regulation of p53 stability mirrored the effects observed in Karpas 299 cells (Figure 5A). Suppression of p53 along with ARNT isoform 1 also rescued the cell proliferation defect with a significant reduction in the expression of *CDKN1A* and a trend of lower *CDKN2B* expression (Figure 5B-5D). Together these findings indicate that ARNT controls p53 in different cell types, which suggests the existence of a general mechanism whereby the ARNT isoforms regulate p53 activity to control cell proliferation.

### ARNT isoform 1 imparts chemo-resistance and survival to cancer cells

**Figure 6:**
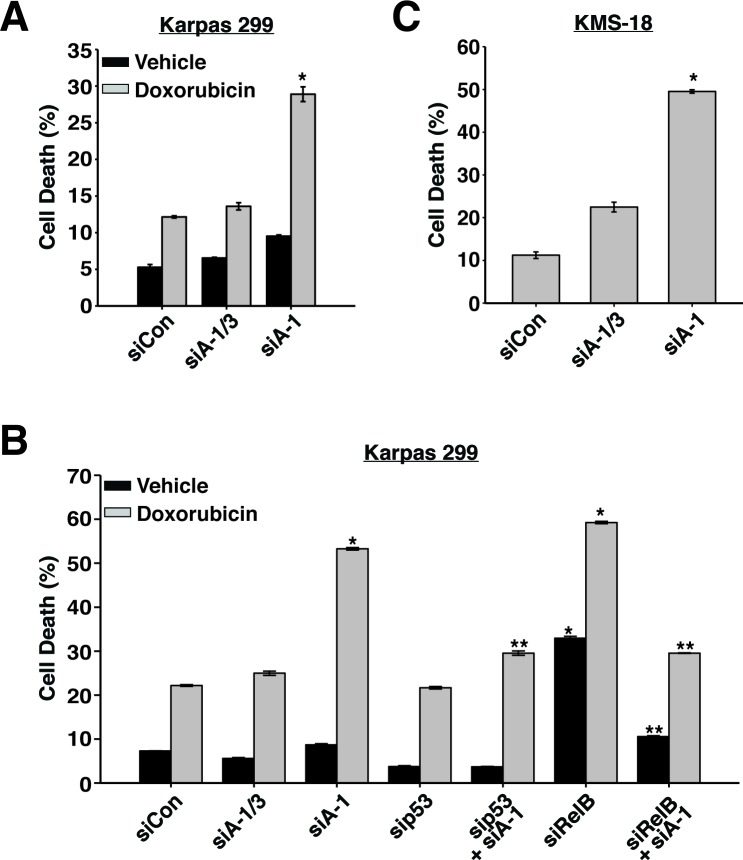
ARNT isoform 1 imparts chemo-resistance and survival to cancer cells **A.** Karpas 299 cells were electroporated with siControl, siA-1/3, or siA-1, and 48 hours later, were treated with vehicle control or 1 μM doxorubicin. Cell death was analyzed 36 hours post-treatment by annexin V staining. **B.** Karpas 299 cells were electroporated with siControl, siA-1/3, siA-1, sip53, sip53 and siA-1, siRelB, or siRelB and siA-1, and 48 hours later, were treated with vehicle control or 1 μM doxorubicin. Cell death was analyzed 36 hours post-treatment by annexin V staining. **C.** KMS-18 cells were electroporated with siControl, siA-1/3, or siA-1, and 64 hours later, cell death was analyzed by annexin V staining. *p < 0.001 compared to siControl. **p < 0.001 compared to siA-1.

Since p53 was stabilized and activated when ARNT isoform 1 was suppressed, we predicted that in the absence of ARNT isoform 1, cells would be highly susceptible to DNA damaging agents. To examine this hypothesis, we lowered ARNT isoform 1 in Karpas 299 cells and treated them with 1 μM doxorubicin. Annexin V staining of these cells revealed that loss of ARNT isoform 1 sensitized cells to doxorubicin as they exhibited an ∼3-fold increase in apoptosis when compared to control cells or cells with reduced levels of both isoforms (Figure 6A and 6B). As expected, co-suppression of p53 with ARNT isoform 1 rescued cells from doxorubicin-induced death (Figure 6B).

Because ARNT coordinates with RelB to regulate different cellular processes, we also examined the role of RelB in cell death caused by doxorubicin exposure. Interestingly, suppression of RelB led to spontaneous apoptosis, which greatly increased after doxorubicin treatment (Figure 6B). Based on these results, we expected that co-suppression of ARNT isoform 1 and RelB would increase the sensitization to cell death. However, to our surprise, reducing RelB and ARNT isoform 1 together rescued the apoptosis that was induced by depletion of either one alone (Figure 6B). This unexpected finding demonstrates that the observed cell death requires RelB or ARNT isoform 1, suggesting redundant functions in controlling apoptosis. Remarkably, suppression of ARNT isoform 1 in KMS-18 cells led to high rates of spontaneous apoptosis in the absence of doxorubicin (Figure 6C). Collectively, our data uncover a mechanism that is governed by ARNT isoform 1 and 3, RelB, and p53 to control the proliferation and survival of cancerous blood cells.

## DISCUSSION

ARNT has been shown to regulate the anti-oxidant response and resistance to chemotherapeutic drugs in diverse cancers and is important for AHR or HIF-1 promoted tumors [13-19]. Whereas these previous reports treat ARNT as a single protein, we have noted that, as compared to primary human B and T cells, various lymphoid malignancy cell lines contain increased levels of ARNT isoform 1 and decreased levels of ARNT isoform 3. Given these observations, we surmised that excessive amounts of ARNT isoform 1 contributed to cancer proliferation and survival. Having previously found cooperation between RelB and ARNT, we examined whether the ARNT isoforms affected RelB activity [20]. Interestingly, simultaneous suppression of the ARNT isoforms greatly stabilized RelB protein whereas decreasing ARNT isoform 1 modestly destabilized RelB protein. Thus, ARNT isoforms control the half-life of RelB, which may occur through alterations in the transcription of genes that regulate RelB stability or by recruiting RelB into an AHR/ARNT cullin 4B ubiquitin ligase complex [40]. While future studies will address these possibilities, our current data support a model where increased ARNT isoform 1 serves to block isoform 3 and RelB from inducing cell cycle arrest.

Intriguingly, RelB has been shown to promote the growth of primary human fibroblast cells, and cells from chronic lymphocytic leukemia (CLL) patients, through regulating p53 stability, EZH2 expression and Rb activation [35]. Our data are consistent with these results as depletion of both ARNT isoforms leads to increased RelB stability and p53 instability whereas, suppression of ARNT isoform 1 results in RelB instability and p53 stability and activation. It may be that ARNT governs p53 stability and activation by modulating RelB protein stability and thereby the expression of genes that control p53 activity. That being said, the instability of RelB is modest after depletion of ARNT isoform 1, pointing to a supporting role for ARNT isoform 3 in the stabilization of p53.

We have noted that a common 1q21 focal amplification in MM and ALCL, which is an indicator of poor outcome for patients, contains the *ARNT* gene [3, 41]. However, this amplification would be predicted to result in higher levels of both ARNT isoforms. Thus, the question arises as to how the levels of ARNT isoforms are differentially changed. Analysis of the *ARNT* epigenetic landscape has provided important clues into possible splicing regulation of alternative exon 5 (Figure S2) [30]. Primary leukocytes exhibit a striking histone modification pattern (H3K27ac, H3K4me1) centered on exon 5 of *ARNT* that is absent from most all other primary tissues and cancer cells. This observation suggests that *ARNT* expression may be uniquely regulated in hematopoietic cells, which is supported by the relative sensitivity of the immune system to xenobiotic exposure and the dependence of adult and fetal hematopoietic stem cells on ARNT for viability and homeostasis [42, 43]. Although H3K27ac and H3K4me1 are well-characterized histone marks associated with promoters and enhancer regions, there is another possibility to consider [44, 45]. It is now accepted that alternative splicing of pre-mRNA occurs cotranscriptionally in a manner that is directed by recruitment of the splicing machinery to specific histone marks [46, 47]. Furthermore, histone marks affect the elongation rate of RNA polymerase II, and the elongation rate can dictate whether or not an alternative exon is included in the processed mRNA [47]. A slow rate leads to increased inclusion of an alternative exon whereas a faster elongation rate of RNA polymerase II, which occurs when there is a more relaxed chromatin structure, such as hyperacetylated chromatin, causes skipping of the alternative exon. While future investigation is needed to fully understand the importance of these histone marks that surround exon 5, it is reasonable to predict that in primary B and T cells alternative exon 5 would be skipped more readily whereas, in cancers that have lost the H3K27ac and H3K4me1 marks, alternative exon 5 would be included more often. Importantly, these predictions based on the Epigenome Roadmap data are supported by a previous study and by our immunoblot analysis of ARNT isoform levels in primary B and T cells versus various lymphoid malignancies (Figure 1) [48].

Altogether, our data demonstrate that suppressing ARNT isoform 1 ultimately activates p53, leading to augmented expression of cell cycle inhibitors, such as *CDKN1A*, and S phase cell cycle arrest. These observations indicate that ARNT isoform 1 is a promising candidate to target therapeutically. Splice modulation therapy is an attractive option for depleting ARNT isoform 1, which allows for exon skipping by a modified antisense oligonucleotide (AONs) that hybridizes to complementary sequences in the target exon and hides it from the splicing machinery [49]. Successful clinical trials have shown promise for using AONs to treat Duchenne muscular dystrophy [50]. Moreover, AONs have been used to switch STAT3 isoforms in cancer cells to promote apoptosis and inhibit tumor growth [51]. Therefore, targeting exon 5 in the pre-mRNA of ARNT would theoretically bolster the production of ARNT isoform 3, which would presumably activate p53 more robustly than specifically targeting ARNT isoform 1 for depletion. Importantly, depleting all ARNT isoforms with a common RNAi approach would be predicted to benefit cancer proliferation in the lymphoid malignancies examined. This hypothesis is supported by our data in this study, and by the observation that a rare pediatric CLL patient was negative for frequently associated genetic abnormalities but exhibited a deletion of the *ARNT* gene [52]. Overall, our results suggest that ARNT isoform 1 and 3 contain differential activities and that variations in isoform ratios lead to differential phenotypes. In conclusion, our findings underscore the need for careful evaluation of unique ARNT isoform activities and how they collectively regulate the multitude of cellular functions associated with ARNT signaling.

## MATERIALS AND METHODS

### Cell culture

OPM2, NCI-H929, KMS-11, KMS-11 adherent, KMS-18, and L363 were obtained from P. Leif Bergsagel. Normal human B and T cells were isolated by magnetic selection (Miltenyi Biotec) from leukocyte samples obtained from unidentified healthy volunteers through the New York Blood Center. KMH2, Raji, Karpas 299, Jurkat, and L540 cell lines were obtained from C. Duckett (University of Michigan Medical School, Ann Arbor). All lymphoid cells and lymphoid cancer cells were cultured in RPMI-1640 (Corning) containing 10% fetal bovine serum (Gemini) and 2 mM glutamine (Life Technologies) at 37°C and 5% CO_2_. HEK 293T cells were cultured in DMEM containing 10% FBS and 2 mM glutamine at 37°C and 5% CO_2_.

### ARNT copy number analysis

HMCLs were analyzed by aCGH on the Agilent 44k platform. The aCGH data was segmented using CBS algorithm as described [23]. Data was supplied by P. Leif Bergsagel (Mayo Clinic, Scottsdale, AZ) and is available at the MMRC genomics portal (http://www.broadinstitute.org/mmgp/home).

### RNA interference

Cells were electroporated with 2 μM of target siRNA duplexes as previously described [38]. Double RNAi experiments were normalized with control siRNA. Ficoll-Paque spins were performed at 16 hours post-transfection and cells were transferred to RPMI-1640 medium containing 10% FBS and 2 mM glutamine at 0.5 × 10^6^ cells/mL until analyzed. The target sequence for siControl is a scrambled siARNT-1/3 sequence and both have been described [20]. The target sequences (Sigma) are as follows: siARNT-1 5′-UGC CAG GUC GGA UGA UGA GCA-3′; siRelB 5′-GAC UGC ACC GAC GGC AUC U-3′; siRelA 5′-GCC CUA UCC CUU UAC GUC-3′; and sip53 5′-CCG GAG GCC CAU CCU CAC C-3′.

### Antibodies and immunoblotting

Whole cell lysates were prepared by incubating cells for 20 minutes on ice in radioimmune precipitation assay (RIPA) buffer (PBS containing 1% Nonidet P-40, 0.5% [w/v] deoxycholic acid, 0.1% SDS, 1 mM PMSF, and 1 mM DTT) supplemented with complete mini protease inhibitor tablets (Roche), 0.2 mM sodium orthovanadate, and 50 mM sodium fluoride. Lysates were analyzed by immunoblotting as previously described [53]. Antibodies used were: ARNT (BD Pharmingen); β-actin (Sigma); RelB and RelA (Santa Cruz); p53 (Invitrogen); HRP-conjugated p53 and phosphorylated p53^S15^ (R&D Systems).

### Cell proliferation analysis, quantitative PCR, cell cycle analysis, and protein stability

For cell proliferation analysis, Karpas 299 and KMS-18 cells were electroporated with the indicated siRNAs. Twenty-four hours after transfection, cells were placed at 1.7 × 10^5^ cells/mL (Karpas 299) and 0.5 × 10^6^ cells/mL (KMS-18) per well in 6-well plates in triplicate. Cells were counted at 24-hour intervals for a total of 72 hours or 96 hours using a Z2 Beckman Coulter Particle Count and Size analyzer. Quantitative PCR and cell cycle analysis were performed as previously described [53]. To assess protein half-life, cycloheximide (Sigma) was added to cells (electroporated with the indicated siRNAs) at 100 μg/mL 60 hours post-transfection. Cells were harvested at the indicated time points and analyzed by immunoblotting.

### Cell death analysis

Cells were pelleted at 300 × g for 5 minutes, washed with PBS, and resuspended in 1 mL annexin V binding buffer (10 mM HEPES, pH 7.4, 140 mM NaCl, and 2.5 mM CaCl_2_). Cells were incubated with 100 ng/mL Annexin-FITC for 8 minutes and PI was added just prior to flow cytometry analysis.

### Statistical analysis

All experiments were performed at least 3 times, in triplicate. Data are graphed as mean ± s.e.m. Quantitative PCR experiments are shown as one representative experiment. Cell cycle and cell death data were analyzed using FlowJo (Tree Star Inc.). Statistical significance was determined by student's t-test. For non-normal data, or data with significantly different variances, the non-parametric Mann-Whitney Ranks Sum test was used. Results were considered significant when *p* < 0.05.

## SUPPLEMENTARY MATERIAL FIGURES


