# Supplementary material for: Aryl hydrocarbon receptor nuclear translocator (ARNT) isoforms control lymphoid cancer cell proliferation through differentially regulating tumor suppressor p53 activity
Source: Oncotarget. 2016 Feb 20;7(10):10710–22. doi: 10.18632/oncotarget.7539 (PMC4905433; doi:10.18632/oncotarget.7539)
Supplement: Supplementary file 1 [file oncotarget-07-10710-s001.pdf]

Aryl hydrocarbon receptor nuclear translocator (ARNT) isoforms control lymphoid cancer cell proliferation through differentially regulating tumor suppressor p53 activity

Supplementary Material

A

SAMPLE	LOCUS	COPY NUMBER
ANBL6	*ARNT*	2.180088282
ARP1	*ARNT*	4.577002048
CAG	*ARNT*	5.570586205
DELTA47	*ARNT*	5.709896564
EJM	*ARNT*	2.310377598
FLAM76	*ARNT*	2.126859426
FR4	*ARNT*	4.735713959
H1112	*ARNT*	2.823943138
INA6	*ARNT*	2.428283453
JIM3	*ARNT*	8.066644669
JJN3	*ARNT*	3.354818106
JK6L	*ARNT*	4.207172871
KARPAS620	*ARNT*	2.560871601
KHM11	*ARNT*	5.286267757
KHM1B	*ARNT*	7.544189453
KMM1	*ARNT*	2.841897726
KMS11	*ARNT*	3.987119675
KMS12BM	*ARNT*	2.827967167
KMS12PE	*ARNT*	3.253892899
KMS18	*ARNT*	3.421411276
KMS20	*ARNT*	6.815284252
KMS26	*ARNT*	2.981743097
KMS28BM	*ARNT*	3.274240494
KMS28PE	*ARNT*	3.511126518
KMS34	*ARNT*	2.48257041
L363	*ARNT*	2.905746937
LP1	*ARNT*	4.724547863
MM1R	*ARNT*	3.193591356
MM1S	*ARNT*	3.315104723
MM_M1	*ARNT*	4.242246628
NCIH929	*ARNT*	4.868062496
OCIMY1	*ARNT*	2.045366049
OCIMY5	*ARNT*	1.985526204
OCIMY7	*ARNT*	3.692774534
OPM1	*ARNT*	3.624837637
OPM2	*ARNT*	5.21121788
PE1	*ARNT*	2.668722868
PE2	*ARNT*	2.939680099
RPMI8226	*ARNT*	3.811626434
SACHI	*ARNT*	2.053567886
SKMM1	*ARNT*	4.144442558
SKMM2	*ARNT*	2.115360737
U266	*ARNT*	3.01539278
UTMC2	*ARNT*	3.401702642
XG1	*ARNT*	2.899944782
XG2	*ARNT*	2.990574598
XG6	*ARNT*	3.940377474
XG7	*ARNT*	5.152966022

B

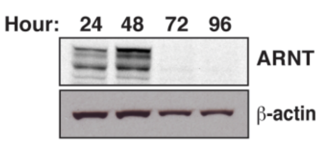

C

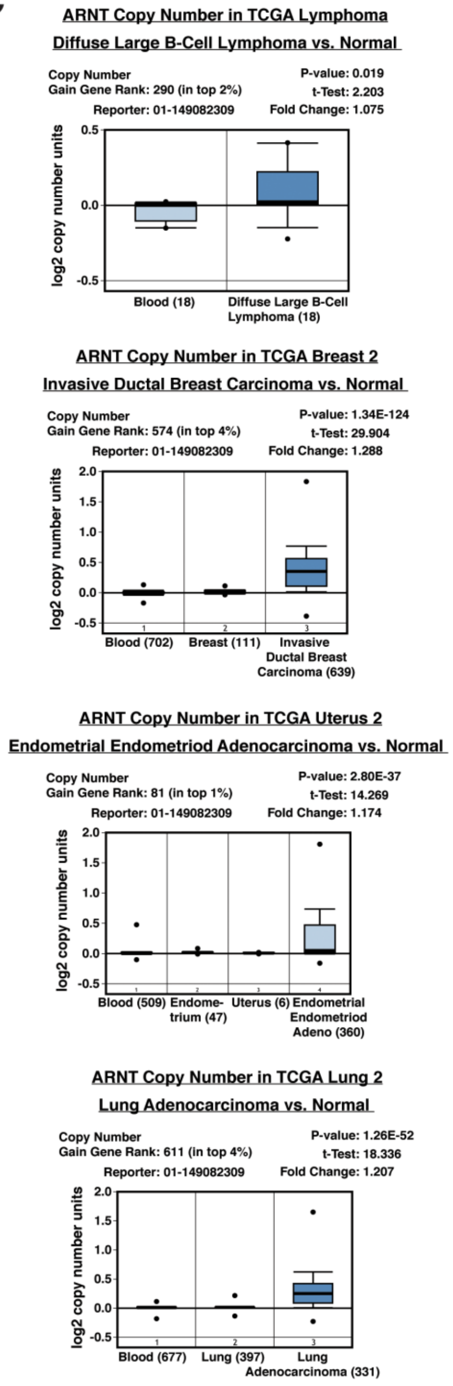

Supplemental Figure 1: Shown in this figure is expanded ARNT copy number analysis of human myeloma cell lines and Oncomine analysis of ARNT copy number in various cancer types.

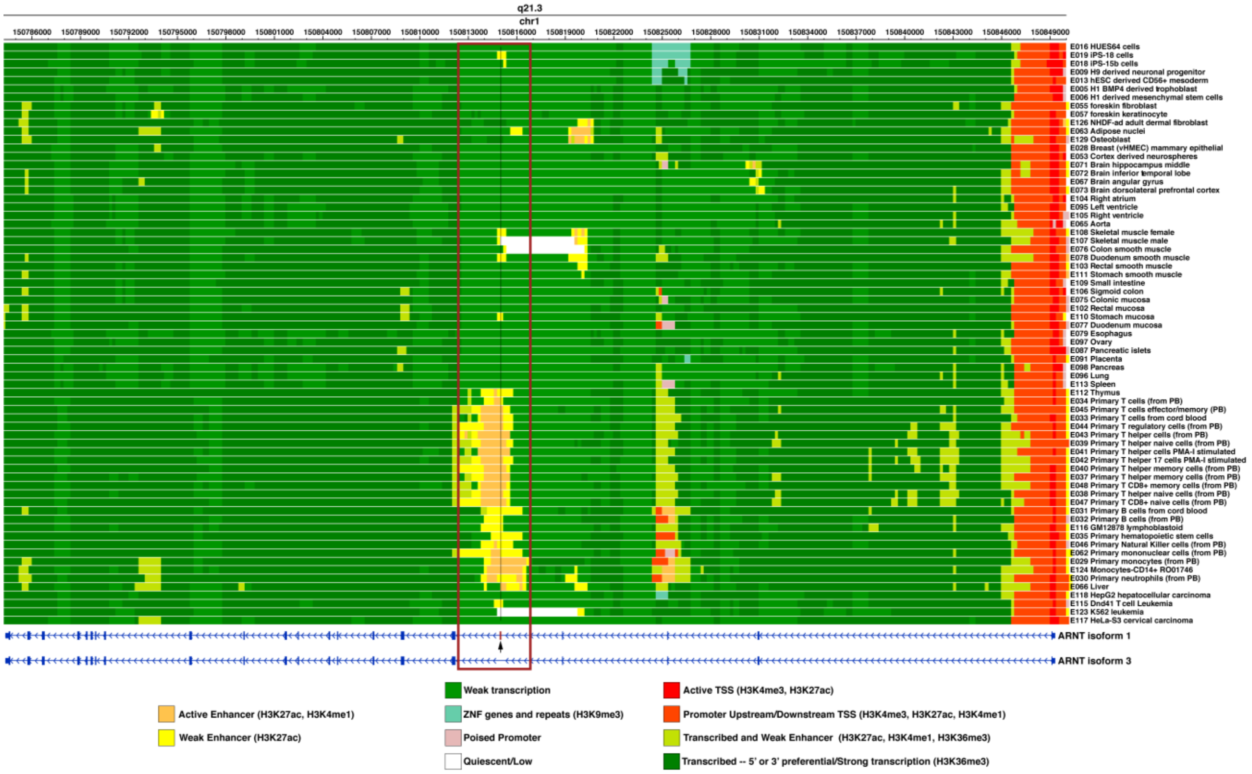

Supplemental Figure 2: Epigenomic profiles of the ARNT locus obtained from data sets from the NIH Roadmap Epigenomics Consortium, indicating a unique difference between histone modifications between primary lymphoid cells and cancer cells.
